# Torrefaction of Demineralized Wood with Flue Gas: Kinetics, Product Distribution, and Thermal Conversion

**DOI:** 10.3390/polym18111370

**Published:** 2026-05-31

**Authors:** Xiaoyu Zhang, Jingkun Han, Shan Cheng, Hong Tian, Jing Gu, Xiaoteng Jiang

**Affiliations:** 1 College of Energy and Power Engineering, Changsha University of Science & Technology, Changsha 410114, China; zxiaoyu2026@126.com (X.Z.);; 2 Guangzhou Institute of Energy Conversion, Chinese Academy of Sciences (CAS), Nengyuan Road, Guangzhou 510640, China; 3 Guangdong Provincial Key Laboratory of High-Quality Recycling of End-of-Life New Energy Devices, Guangzhou 510640, China

**Keywords:** torrefaction, flue gas, demineralized wood, kinetics, thermal conversion

## Abstract

Flue gas torrefaction is an emerging biomass pretreatment technology that utilizes industrial flue gas as a reactive medium to replace inert atmospheres. However, the intrinsic complexity of biomass and the catalytic interference of ash hinder mechanistic elucidation. This study investigated the torrefaction behavior of demineralized poplar wood under N_2_, CO_2_, dry flue gas (DFG), and wet flue gas (WFG) at 300 °C for 5–20 min. Thermogravimetric analysis combined with kinetic modeling (FWO, KAS, and CR methods) revealed that the apparent activation energy (*E_α_*) varied non-monotonically with atmosphere oxidizability. Under N_2_, the average *E_α_* was 177 kJ/mol following the three-dimensional diffusion model (D5). CO_2_ gave the highest average *E_α_* (314 kJ/mol) with the Avrami–Erofeev nucleation model (A_1_/_4_). DFG and WFG significantly reduced the average *E_α_* to 133 and 128 kJ/mol, respectively, both following the A_1_/_3_ model. Consistently, WFG yields the lowest char and the highest gas yield. XPS and FTIR analyses indicated that flue gas atmospheres, especially WFG, promoted deeper deoxygenation and aromatization of biochar. Tar composition underwent a noticeable transition from ketones to aldehydes and saccharides under flue gas conditions, with the most remarkable variation observed under WFG. Gaseous products were dominated by CO_2_ under N_2_ and by CO under CO_2_, while DFG and WFG produced moderate and stable gas compositions. These findings demonstrate that flue gas torrefaction, particularly under WFG, effectively enhances biomass effectively upgrades biomass quality by regulating pyrolysis kinetics and product distribution, and demineralized biomass is a suitable intermediate model for mechanistic investigation.

## 1. Introduction

To achieve global sustainable development, nearly 70% of countries and regions worldwide have proposed carbon neutrality targets around 2050 [1]. Accordingly, the development and utilization of clean renewable energy have become increasingly imperative [2]. Biomass energy, as a carbon-neutral renewable resource with wide geographical distribution, possesses great application potential in future energy systems [3,4,5,6].

However, raw biomass suffers from inherent drawbacks, including high moisture content, low energy density, strong hydrophilicity, poor grindability, and easy biodegradability. These shortcomings lead to high transportation and storage costs and severely restrict large-scale industrial application efficiency. To address these limitations, various biomass pretreatment technologies have been developed, among which torrefaction is regarded as one of the most promising and commercially viable thermochemical pretreatment methods [7]. Torrefaction can effectively improve the energy density, hydrophobicity, and grindability of biomass through a low-temperature (200–300 °C) pyrolysis process, thereby solving technical and economic issues in its processing, storage, and transportation. In addition, the combustion characteristics of torrefied biomass are comparable to lignite. For coal-fired power plants co-firing biomass, torrefaction can significantly increase the biomass blending ratio without deteriorating boiler operation efficiency and load stability. However, conventional torrefaction requires continuous heat supply and N_2_ injection, which greatly increases the process cost [8,9,10]. Moreover, it is difficult to integrate independent torrefaction systems into existing coal-fired units, which greatly limits their large-scale commercial promotion [11].

Flue gas torrefaction is an advanced biomass pretreatment method designed to address the constraints of conventional torrefaction processes. Its core concept is to replace expensive inert gas (e.g., N_2_) with industrial boiler exhaust flue gas. Typical flue gas consists mainly of N_2_, CO_2_, O_2_, and steam (H_2_O) discharged from coal or biomass combustion facilities. Compared with conventional N_2_ torrefaction, flue gas torrefaction presents the following advantages: (1) flue gas generally contains over 70% N_2_, eliminating the cost of N_2_ production and separation in traditional torrefaction; (2) flue gas has a certain temperature, which can reduce the heat supply during torrefaction; (3) it can be easily coupled with existing heat and power generation systems to realize the integration of biomass upgrading and utilization. Meanwhile, flue gas torrefaction exhibits favorable performance in improving the physicochemical properties, combustion and pyrolysis characteristics of biomass, and reducing NO_x_ emissions during thermal conversion [12]. This waste-to-resource utilization mode significantly lowers biomass pretreatment costs and environmental impacts, while providing a new pathway for flue gas resource recycling, which is highly consistent with circular economy and sustainable development concepts [13,14].

From a technical perspective, flue gas torrefaction is not merely a simple atmospheric replacement. Instead, reactive components in flue gas participate in complex physicochemical interactions with biomass during pyrolysis [13,15]. For instance, O_2_ and H_2_O facilitate the cleavage of ether bonds and C–C bonds in biomass, while CO_2_ may undergo Boudouard reactions with newly formed active surfaces after biomass pyrolysis. Therefore, flue gas torrefaction is a complex process integrating physical drying, pyrolysis, and partial gasification and oxidation. On the other hand, as a natural polymer composite composed of cellulose, hemicellulose, and lignin polymers, the inherent complexity of biomass components poses significant challenges to the study of its thermochemical conversion processes. Previous research on biomass torrefaction in flue gas atmospheres has primarily followed two approaches. The first uses model compounds such as cellulose, lignin, etc., which have well-defined compositions and facilitate mechanistic investigations. However, these model systems are far simpler than real biomass, neglecting component interactions and the potential effects of minor constituents, leading to reaction behaviors that may differ fundamentally from those in actual systems. The second approach directly employs real biomass, which, despite reflecting the complexity of practical feedstocks, inevitably contains ash and inorganic elements that can catalyze or inhibit key reaction pathways. This renders the reaction system overly complicated, making it difficult to effectively isolate and elucidate intrinsic reaction processes. Thus, model compound systems lack representativeness, while real biomass systems introduce excessive interfering factors. Neither approach independently supports an in-depth understanding of the key reaction processes in flue gas torrefaction. Therefore, there is an urgent need to establish an intermediate research system that bridges model compounds and real feedstocks by controlling system complexity while preserving the key structural features of biomass [16,17].

To address this issue, this study adopts demineralized biomass as the research object to investigate its thermochemical conversion behavior during flue gas torrefaction. Acid washing is employed to remove alkali and alkaline earth metals from the ash, preserving the native organic skeleton of the biomass. Kinetic modeling is performed using thermogravimetric analysis to reveal the intrinsic reaction mechanisms. Combined with systematic physicochemical characterization of the three-phase products (char, tar, and non-condensable gas), the study elucidates the biomass evolution during torrefaction and the role of the flue gas components.

## 2. Materials and Methods

### 2.1. Biomass Pretreatment and Properties

Poplar wood powder was used as the raw material. Before the experiment, the poplar wood powder was crushed and sieved to 100–200 mesh, then dried to constant weight under 105 °C. The demineralization process was as follows: 50 g dried poplar powder was immersed in 0.5 mol/L sulfuric acid solution and magnetically stirred at 60 °C for 12 h. The solid residue was filtered, rinsed with deionized water until pH reached 7.0 ± 0.1, and dried to a constant weight at 105 °C. According to the GB/T 28731-2012 standard [18], the proximate analysis of the raw and demineralized biomass was performed. As shown in Table 1, the biomass is mainly composed of volatile matter. After acid leaching, the ash content decreases to 0.03%, indicating efficient demineralization.

### 2.2. Torrefaction Tests

**Figure 1:**
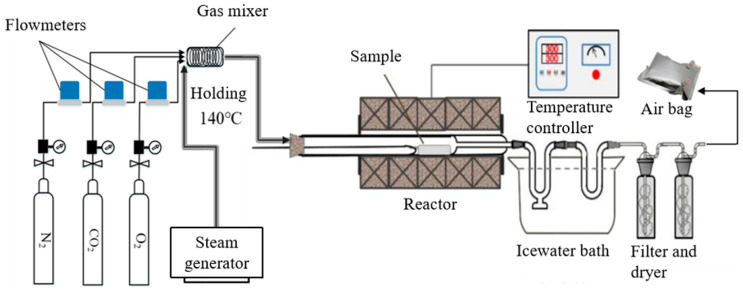
Schematic diagram of the tube furnace experimental system.

The torrefaction tests were carried out with a vertical fixed-bed reactor system, as shown in Figure 1. Prior to the experiment, carrier gas was purged into the reactor to remove residual air. When the reactor temperature reaches the set temperature of 300 °C, ~1 g sample in the quartz boat is quickly fed into the reactor center, and heated for 5, 10, and 20 min. The investigated atmospheres were N_2_, CO_2_, dry flue gas (DFG) simulated by a mixture of 80% N_2_, 15% CO_2_, and 5% O_2_, and wet flue gas (WFG) simulated by a mixture of 70% N_2_, 15% CO_2_, 5% O_2_, and 10% H_2_O. The H_2_O was provided by a steam generator filled with deionized water. The steam circuit was maintained at 140 °C by electric heating to avoid condensation. These gases were introduced into the reactor at a flow rate of 50 mL/min, which is precisely controlled through mass flowmeters (±1% accuracy). The solid torrefied char was collected after quenching. The tar was collected using isopropanol from the condenser tube. The non-condensed gas was collected by the air bag.

### 2.3. TG Analysis and Kinetic Modeling

#### 2.3.1. TG Tests

TG analysis was performed using a thermogravimetric analyzer (STA449F3 Jupiter, NETZSCH, Selb, Germany). During the experiment, approximately 10 mg of the sample was accurately weighed and placed in a ceramic crucible. The experiments were conducted under N_2_, CO_2_, DFG, and WFG with a flow rate controlled at 80 mL/min. The heating rates were set as 5, 10, and 15 K/min, with a final heating temperature of 500 °C. To ensure the reliability of the data, each experimental condition was repeated three times, and the resulting thermogravimetric curves and characteristic point data were statistically analyzed.

#### 2.3.2. Flynn-Wall-Ozawa (FWO) Method

The FWO method can obtain the apparent activation energy (*E_α_*) by plotting the natural logarithm of ln*β_i_* against 1000/*T_αi_*, which shows a linear relationship between different heating rates and specific conversion rate [19,20].

(1) $$\ln\left(\beta_{i}\right)=\ln\left(\frac{A_{\alpha }E_{\alpha }}{R_{g}\left(\alpha \right)}\right)-5.331-1.052\frac{E_{\alpha }}{RT_{\alpha i}}$$

where g(α) is a constant at a given conversion rate. The subscripts *i* and *α* represent the specified heating rate and the specified conversion rate, respectively. The activation energy *E_α_* is calculated from the slope −1.052*E_α_*/*R*.

#### 2.3.3. Kissinger–Akahira–Sunose (KAS) Method

The KAS method is based on the following expression [21,22]:

(2) $$\ln\left(\frac{\beta_{i}}{T_{\alpha i}^{2}}\right)=\ln\left(\frac{A_{\alpha }R}{E_{\alpha }g\alpha }\right)-\frac{E_{\alpha }}{RT_{\alpha i}}$$

The apparent activation energy can be obtained by plotting the relationship curve between ln*β_i_*/*T^2^_αi_* and 1000/*T_αi_*, corresponding to a given conversion rate *α*, with the slope equal to −*E_α_*/*R*.

#### 2.3.4. Coats–Redfern (CR) Method

The CR method is a typical model-fitting approach used to determine the reaction mechanism and kinetic parameters by fitting experimental thermogravimetric data to theoretical model functions. Unlike isoconversional methods such as FWO and KAS, the CR method assumes a specific reaction model g(α) and calculates the corresponding kinetic parameters directly from a single heating rate experiment or by combining multiple heating rates [23,24].

The CR method is expressed by the following equation:

(3) $$\ln\left(\frac{{g(\alpha )}_{i}}{T_{\alpha i}^{2}}\right)=\ln\left(\frac{A_{\alpha }R}{E_{\alpha }\beta_{i}}\right)-\frac{E_{\alpha }}{RT_{\alpha i}}$$

where *g*(*α*) is the integral form of the reaction mechanism function, *T_αi_* is the absolute temperature at a given conversion rate α and heating rate *β_i_*, *A_α_* is the pre-exponential factor, and *R* is the universal gas constant.

### 2.4. Characterization of the Torrefaction Products

The composition of the non-condensable gases was measured using a gas chromatograph (GC, Agilent 7890A, Cork, Ireland). The composition of tar was measured using a gas chromatography-mass spectrometry instrument (GC/MS, Thermo Fisher, TRACE 1300ISQ, Milan, Italy) [25]. The BET specific surface area of the solid samples was measured with N_2_ adsorption isotherms at −196 °C on an automated surface area and pore size analyzer (Quantachrome, Autosorb-iQ-2, Boynton Beach, FL, USA). The surface functional groups of char were characterized by Fourier-transform infrared spectroscopy (FTIR, Nicolet iS50, Madison, WI, USA) and X-ray photoelectron spectrometer (Thermo Fisher, ESCALAB 250Xi, East Grinstead, UK).

## 3. Results and Discussion

### 3.1. Thermal Degradation Characteristics and Kinetic Analysis

#### 3.1.1. TGA Results

**Figure 2:**
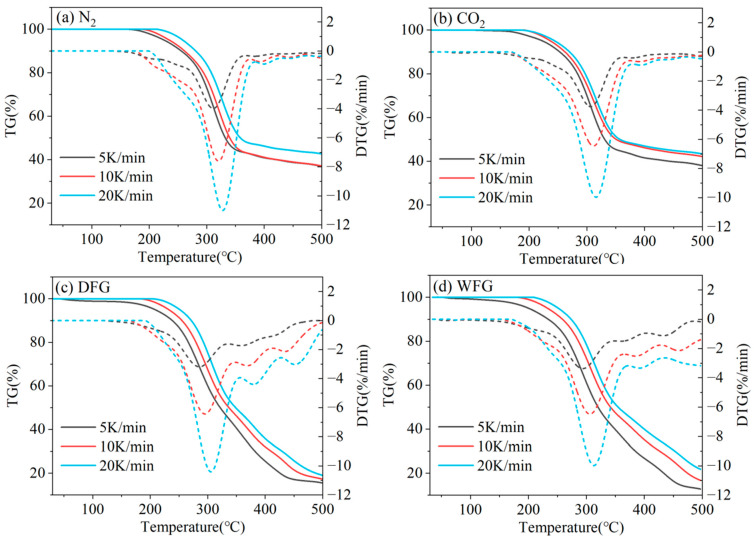
TG and DTG curves under different atmospheres and heating rates. (solid lines for TG; dashed lines for DTG).

Figure 2 shows the TG and differential thermogravimetric (DTG) curves of the biomass pyrolysis under different atmospheres and heating rates. The thermal degradation process can be divided into three distinct regions corresponding to moisture evaporation, active pyrolysis, and passive pyrolysis stages. The first stage (100–150 °C) corresponds to the removal of free and adsorbed water, which is insensitive to atmospheric conditions. The main pyrolysis process (active pyrolysis) occurs approximately at 250–450 °C, with a prominent mass loss peak corresponding to the rapid decomposition of hemicellulose, cellulose, and lignin. Sharp DTG peaks were observed under N_2_ and CO_2_, indicating concentrated pyrolysis behavior. In contrast, DFG and WFG broadened the main DTG peak and generated secondary high-temperature peaks, suggesting that reactive atmospheres promote secondary pyrolysis reactions. As the heating rate increases, the thermal hysteresis effect intensifies, shifting the main peak to higher temperatures, reflecting the transition from kinetic control to diffusion control, consistent with previous reports [26,27,28]. In the passive pyrolysis stage, mass loss under DFG and WFG was significantly higher than that under inert atmospheres, confirming the participation of reactive gasification and oxidation reactions.

An increase in heating rate shifted both TG and DTG peaks to higher temperatures and increased the residual mass in the high-temperature region (15 > 10 > 5 K/min), which is attributed to the hindered release of volatiles under rapid heating. At the same heating rate, the residual mass followed the order: N_2_ > CO_2_ > DFG > WFG, indicating that reactive atmospheres promote char consumption, with WFG exhibiting the strongest reactivity. The DTG curves show that under reactive atmospheres, the peak mass loss rate is higher than that under N_2_, and the peak temperature shifts to lower values, approximately 20–30 °C lower under WFG than under N_2_, suggesting that H_2_O and O_2_ reduce the apparent activation energy of pyrolysis, accelerating volatile release and char gasification. Furthermore, under DFG and WFG atmospheres, the DTG curves exhibit a double-peak feature in the high-temperature region, which may be related to the stepwise decomposition of components or secondary gas-phase reactions in the reactive atmosphere [29,30,31].

#### 3.1.2. Kinetics Analysis

**Figure 3:**
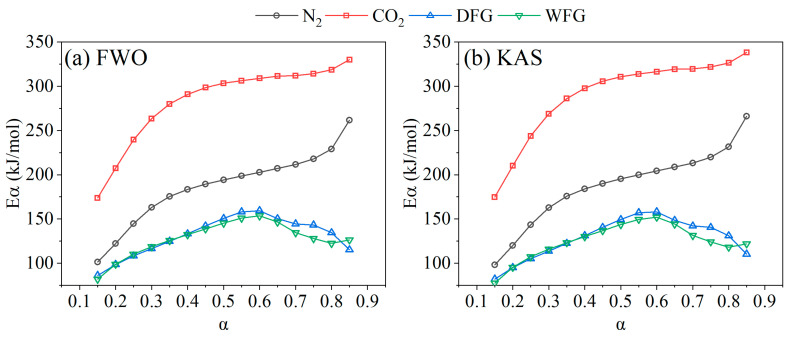
Variation in E_α_ with α calculated by the (**a**) FWO and (**b**) KAS methods.

Kinetic parameters obtained via FWO, KAS, and CR methods were calculated according to Equations (1)–(3), with the conversion rate α given. The FWO plots of ln(*β_i_*) and 1000/*T_αi_* at different conversion rates are shown in Figure S1, and the KAS plots of ln*β_i_*/*T^2^_αi_* and 1000/*T_αi_* are shown in Figure S2. The detailed kinetic parameters calculated by the FWO and KAS methods are shown in Tables S1 and S2. In the main text, the variation in activation energy with conversion rate is presented in Figure 3. It is clear that the FWO and KAS methods yield highly consistent Eα trends across all four atmospheres, confirming the reliability of the kinetic analysis.

The *E_α_* exhibits a non-monotonic trend with increasing atmospheric oxidizability. Under N_2_, *E_α_* increases gradually from approximately 100 to 260 kJ/mol with increasing conversion rate, reflecting the transition from the initial release of volatile components to the more energy-demanding decomposition of cellulose and lignin. Under CO_2_ atmosphere, the *E_α_* is significantly higher, ranging from approximately 170 to 330 kJ/mol. This dramatic increase is likely attributed to the endothermic gasification reaction, which requires a high energy barrier and is governed by gas–solid diffusion [32]. In contrast, under DFG atmospheres, the *E_α_* drops sharply to approximately 80–160 kJ/mol, and it first increases and then decreases as the reaction progresses. It can be attributed to the introduction of O_2_, reducing the energy barrier for bond cleavage. Notably, *E_α_* under WFG is generally slightly lower than that under DFG, indicating that H_2_O exhibits a promoting effect on biomass torrefaction.

**Figure 4:**
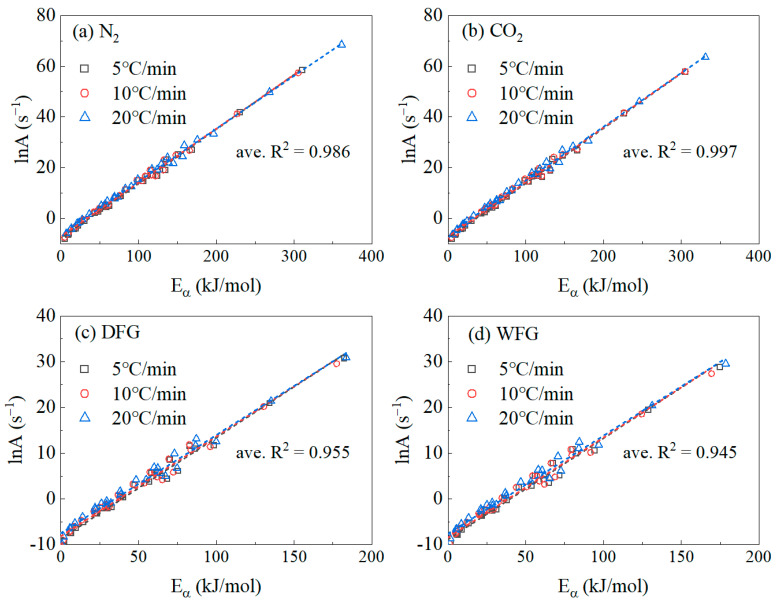
CR dynamic compensation coefficients under four atmospheres.

The CR method was further employed to identify the most probable reaction mechanisms by fitting the experimental data to 29 commonly used reaction model functions, as listed in Table S3. The most appropriate model for each atmosphere was selected based on the highest linear correlation coefficient (R^2^) and the consistency between the calculated CR activation energy and the average obtained from the isoconversional methods [23,24]. The detailed kinetic parameters calculated by the CR method for different atmospheres are listed in Tables S4–S7. The key results are provided in Table 2. Figure 4 shows the compensation coefficients under four atmospheres, calculated based on their corresponding mechanistic models. The good linear fits indicate the models selected are appropriate.

Under N_2_, the best-fitting model was identified as the three-dimensional diffusion model (D5), with an average *E_α_* being 176.95 kJ/mol. It indicates that the torrefaction under N_2_ is governed by three-dimensional diffusion control, where the reaction rate is limited by the diffusion of volatile products within the solid matrix. Under the CO_2_ atmosphere, the optimal model shifted to the Avrami–Erofeev nucleation model (A_1/4_), with an average *E_α_* being 313.93 kJ/mol. This transition from diffusion control to nucleation and growth suggests that the presence of CO_2_ fundamentally alters the reaction pathway, likely by generating new active sites on the char surface. For both the DFG and WFG atmospheres, the Avrami–Erofeev nucleation model (A_1/3_) provided the best fit, while the average *E_α_* is 133.34 kJ/mol for DFG and 128.23 kJ/mol for WFG. It confirms that the O_2_ and H_2_O significantly reduce the reaction barrier and optimize thermal conversion efficiency.

In summary, the combination of isoconversional (FWO/KAS) and model-fitting (CR) methods systematically elucidated the kinetic mechanisms governing the wood torrefaction under different atmospheres. The inert N_2_ was dominated by diffusion control, whereas the reactive flue gas atmospheres shifted the rate-limiting step to nucleation and growth control by altering the reaction pathways. Among all atmospheres, WFG demonstrated the best performance in reducing the reaction energy barrier due to the possible synergistic effect of hydrothermal and oxidative reactions.

### 3.2. Distribution of Multiphase Products from Torrefaction

**Figure 5:**
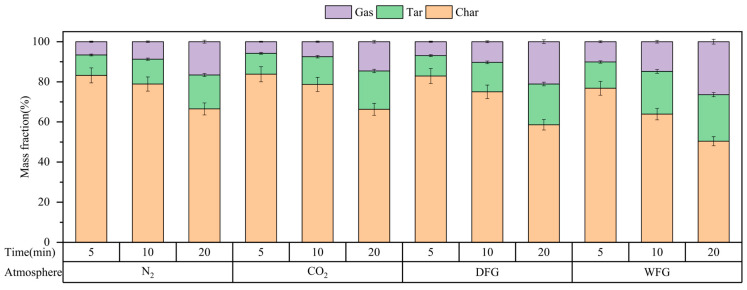
Distribution of the three-phase products from torrefaction.

As shown in Figure 5, the distribution of torrefaction products varies significantly under different atmospheres and times. Overall, char remains the main product, while DFG and WFG produce obviously lower char yield and higher gas yield compared with N_2_ and CO_2_, and WFG yields the lowest char and highest gas content. Additionally, extending the reaction time further reduces char yield and increases gas yield under flue gas, while the time effect is insignificant under inert atmospheres.

This phenomenon is mainly attributed to the gasification and oxidation effects of active components in the flue gas. Water vapor in WFG participates in the water–gas reaction with pyrolytic carbon, while CO_2_ in DFG can be consumed through the Boudouard reaction, both promoting the conversion of solid char into gas. In contrast, N_2_ only provides an inert pyrolysis environment and retains more solid residues [33,34].

WFG exhibits a stronger gasification reactivity than DFG, leading to lower char yields and higher gas yields. This is mainly because water vapor has higher reactivity in gasification reactions than CO_2_ and can also promote secondary cracking of tar at 300 °C. Furthermore, extending the reaction time allows the gasification reactions to proceed more thoroughly, resulting in continuous char reduction and gas accumulation under DFG and WFG, which demonstrates the cumulative effect of gasification reactions.

### 3.3. Effects of Flue Gas Components on Product Characteristics

#### 3.3.1. Char

**Figure 6:**
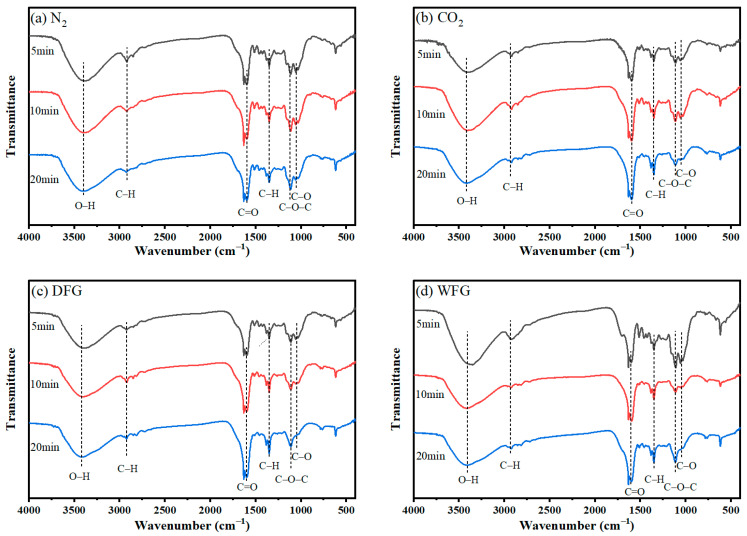
FTIR spectra of the biochars from torrefaction under different atmospheres.

Figure 6 shows the FTIR spectra of the biochars from torrefaction under different atmospheres and times. Under the same atmosphere, as the pyrolysis reaction time extends, the characteristic peak at 1352 cm^−1^ gradually intensifies, while the peak at 1060 cm^−1^ weakens. According to infrared peak attribution, the peak at 1352 cm^−1^ typically corresponds to the bending vibration of C–H bonds in aromatic compounds, and its increasing intensity indicates a rising degree of aromatization during torrefaction. The peak at 1060 cm^−1^ is related to aliphatic C–O bond vibrations, and its weakening reflects the gradual cleavage and decomposition of aliphatic structures during pyrolysis, consistent with the pattern of large organic molecules converting into small molecules in pyrolysis reactions [35]. This trend is consistent with a previous study reporting that torrefaction facilitated the conversion of aliphatic carbon to aromatic rings while reducing C–O and C=O groups in hemicellulose and cellulose [36].

At the same reaction duration, as the atmosphere changes from N_2_ to CO_2_, DFG, and WFG, the peaks at 1115 cm^−1^ and 1060 cm^−1^ show a trend of first weakening then strengthening. The peak at 1115 cm^−1^ corresponds to the stretching vibration of C–O–C bonds in ethers or alcohols, while the peak at 1060 cm^−1^ is attributed to C–OH bond vibrations. This variation may be closely related to the component characteristics of different atmospheres. Specifically, under a CO_2_ atmosphere, the weakened peak intensity suggests that CO_2_ may promote the cleavage of C–O–C and C–OH bonds, possibly through its participation in gasification reactions that consume oxygen-containing functional groups. This observation aligns with the findings of Zhang et al., who reported that CO_2_ gasification enhances the breakdown of oxygenated structures via the Boudouard reaction [15]. In contrast, under DFG and WFG atmospheres, the presence of O_2_ and H_2_O introduces additional oxidative and reforming reactions. O_2_ can facilitate partial oxidation of carbonaceous structures, while H_2_O may enhance hydrolysis and reforming processes, leading to the formation or retention of hydroxyl and ether groups, thereby increasing the corresponding peak intensities. The more pronounced peak recovery under WFG compared to DFG further implies that H_2_O plays a more active role in modifying surface functional groups, likely due to its superior ability to penetrate the biomass matrix and interact with internal active sites.

**Figure 7:**
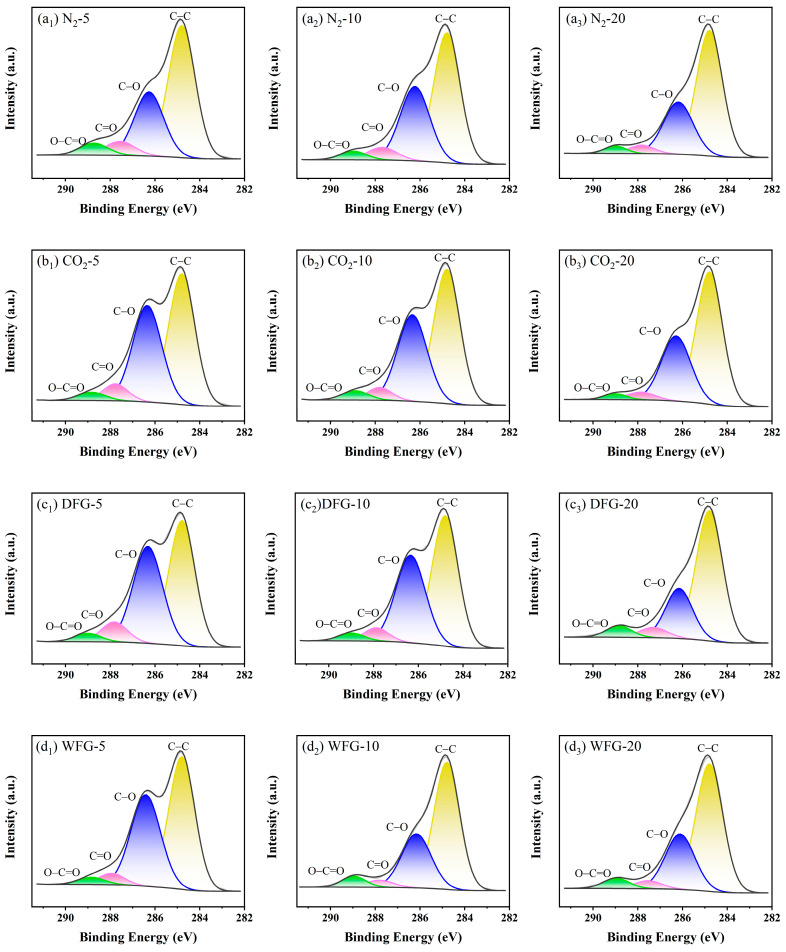
XPS C1s spectra of biochar produced under different atmospheres.

Figure 7 shows the XPS C 1s spectra of the chars, which are deconvolved into four peaks named C_I_ (284.8 eV), C_II_ (286.1–286.3 eV), C_III_ (286.8–287.3 eV), and C_IV_ (288.4–288.8 eV): C_I_ represents the aromatic/aliphatic carbon (C–C, C=C, and C–H); C_II_ represents the C–O/C–O–C groups in the alcohol, ether, and/or phenol; C_III_ represents the carboxyl carbon (C=O and O–C–O); and C_IV_ represents –COO groups in carboxyl and/or ester group. The relative contents of the four kinds of carbon are presented in Table 3. In all samples, C_I_ and C_II_ are the dominant carbon functional groups, with the relative content of C_I_ consistently exceeding 47%, indicating that the carbon skeleton of biochar is mainly composed of stable aromatic rings or aliphatic structures. As the torrefaction time increases, the relative content of C_I_ gradually increases, while that of C_II_ continuously decreases. This trend clearly reflects the occurrence of deep deoxygenation reactions and carbon structural reconstruction within the sample during torrefaction: oxygen-containing functional groups gradually break and escape as small molecules, promoting the transformation of carbon functional groups into lower oxidation states and more stable C–C/C–H structures, ultimately increasing the degree of graphitization or aromatization of the carbon material. This deoxygenation effect during torrefaction has been quantitatively analyzed by Chen et al., who reported that 9.5–63.2% of oxygen in rice husk is removed during torrefaction at 210–300 °C, with H_2_O being the major contributor to deoxygenation, followed by CO_2_ and CO [37]. Although C_III_ and C_IV_ are present in relatively low amounts, their variations also provide important indications. With increasing torrefaction time, the contents of C_III_ and C_IV_ generally decrease, suggesting that carbonyl and carboxyl groups are readily removed under thermal treatment. However, differences are observed among atmospheres. Under CO_2_, the contents of C_III_ and C_IV_ are significantly higher than those under N_2_, which may be attributed to the gasification reaction between CO_2_ and the carbon matrix, generating oxygen-containing intermediates or oxidized surface structures. This is consistent with a recent review on CO_2_-assisted biomass conversion, which noted that CO_2_ integration promotes the Boudouard reaction and influences surface functional group evolution [38]. In contrast, DFG and especially WFG reduce C_III_ and C_IV_ contents, indicating that O_2_ and H_2_O further promote deep deoxygenation and accelerate the cleavage and volatilization of oxygen-containing functional groups [39,40].

Table 4 presents the pore structure parameters of biochar produced under different atmospheres and torrefaction times. Overall, torrefaction exhibits a limited effect on the pore structure. The specific surface area and average pore size increase with time under N_2_. It indicates that prolonging the reaction time under an inert atmosphere facilitates the formation of larger pore structures and enhances the specific surface area. The pore evolution under CO_2_ is more obvious due to continuous gasification etching with reaction time. Under the DFG and WFG atmospheres, the specific surface area also increases with reaction time. Notably, the specific surface areas of the DFG-5 and WFG-5 samples are already higher than those of N_2_-20 and CO_2_-10, indicating that flue gas promotes pore development within a relatively short time. However, the increase in specific surface area slows down with time, suggesting that pore development may enter a plateau phase. At a sufficient reaction time, the presence of O_2_ and H_2_O in the flue gas promotes pore structure development to a certain extent. However, compared to the significant changes in surface chemical properties (e.g., deoxygenation and aromatization), the effect on pore structure is relatively moderate under the experimental conditions. This finding is consistent with the review by Zhang et al., who noted that torrefaction primarily modifies surface chemistry rather than creating extensive porosity under mild conditions [41].

#### 3.3.2. Tar

**Figure 8:**
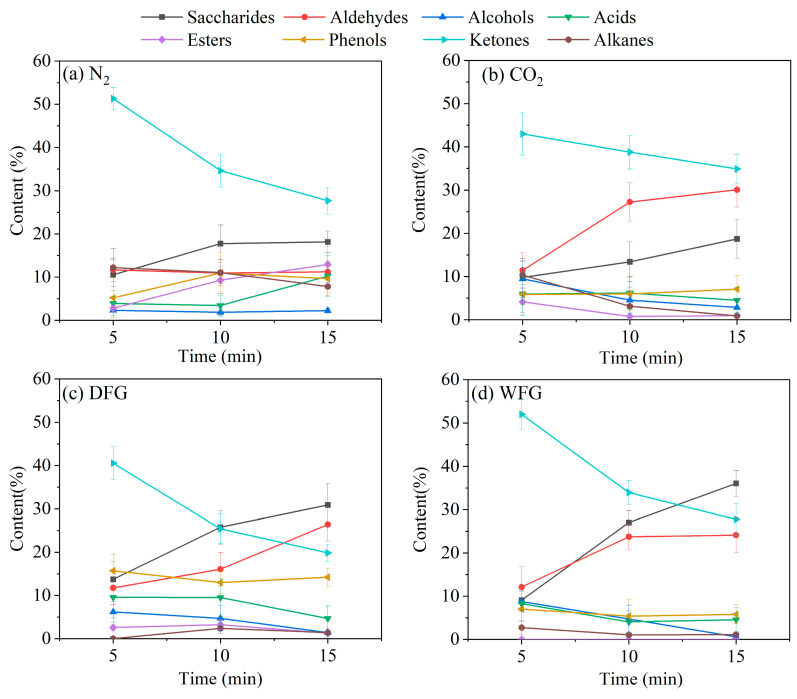
Composition distribution of the torrefied tars over different reaction times.

**Figure 9:**
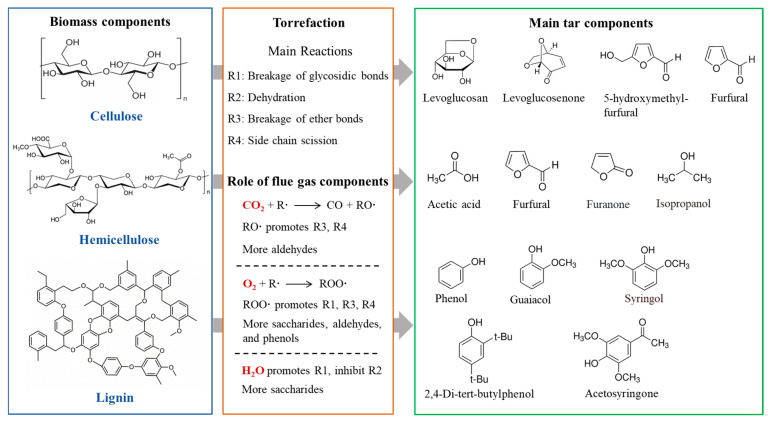
Proposed mechanism of biomass conversion during torrefaction and the effects of the flue gas components on the main tar components.

The tar compositions are shown in Figure 8. Ketones represented by furanone and levoglucosenone display a relatively high initial yield under all reaction conditions, serving as the dominant products during primary pyrolysis. Nevertheless, the relative abundance of ketones decreases continuously with prolonged reaction time across all atmospheres, implying that ketones act as reactive intermediates prone to further cracking or polymerization under extended heating. In contrast, the proportions of aldehydes and saccharides, mainly corresponding to furfural, 5-hydroxymethyl-furfural (5-HMF) and levoglucosan, rise notably with increasing residence time, particularly when the reaction duration is extended from 5 min to 20 min. This trend establishes a clear temporal correlation between the depletion of ketones and the enrichment of aldehydes and saccharides, consistent with previous findings that ketones participate as critical intermediate species in the sequential conversion of biomass polymer components [38]. Combined with the results above, the possible mechanism of biomass conversion during torrefaction and the effects of the flue gas components on the main tar components are proposed, as shown in Figure 9.

Reaction atmosphere imposes a pronounced regulatory effect on the product distribution. Under a N_2_ atmosphere, almost no active radical reactions occur, and the whole thermal conversion proceeds gently with a slow variation in tar components. Compared with N_2_, CO_2_ exerts obvious modulation effects on thermal conversion behavior and product evolution. CO_2_ can react with free radicals to generate RO· radicals and effectively promote the breakage of ester bonds (R3) and side chain scission reaction (R4) of the biomass polymers, resulting in a sharp reduction in esters and more production of aldehydes (e.g., 5-HMF and furfural) [38]. Nevertheless, its regulating intensity is still weaker than that of flue gas atmospheres. In the DFG atmosphere, the ketone content sharply drops from about 41% at 5 min to 20% at 20 min, while aldehydes and saccharides increase substantially. The kinetic analysis reveals that under DFG, the activation energy decreases significantly, and the mechanism becomes A_1_/_3_ nucleation and growth. The lower energy barrier, combined with the presence of O_2_, likely initiates radical-mediated reactions that enhance glycosidic bond cleavage (R1), ether bond breakage (R3), and side chain scission (R4), thereby resulting in enrichment of levoglucosan, furfural, 5-HMF, etc. Similar observations have been reported in oxidative torrefaction studies, where O_2_ was found to promote deeper conversion of tar intermediates. Zhu et al. also reported that higher O_2_ content in flue gas exacerbates cellulose and hemicellulose destruction, leading to more severe torrefaction and altered product distribution [42,43]. WFG results in a higher saccharide yield compared to DFG. It can be attributed to the fact that the additional H_2_O further enhances the cleavage of glycosidic bonds (R1) while inhibiting the dehydration reaction (R2), which promotes the production of levoglucosan and reduces its transformation to levoglucosenone. Overall, the introduction of reactive flue gas components enables more efficient and directional tuning of product distribution via targeted regulation of the radical reaction network and bond cleavage pathways of biomass components.

#### 3.3.3. Gas

**Figure 10:**
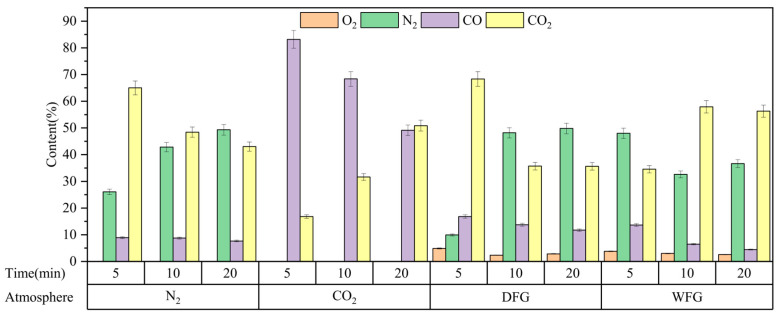
Gas composition of products under different atmospheres and reaction times.

Figure 10 illustrates the volume fraction distribution of gaseous products from torrefaction. Atmosphere and reaction time significantly regulate gas composition with different evolutionary rules. Under N_2_, gas products are dominated by CO_2_, while N_2_ content gradually increases and CO decreases with time. At 5 min, CO_2_ accounts for ~65%, N_2_ for ~26%, and CO for ~9%; at 20 min, CO_2_ decreases to ~43%, N_2_ rises to ~49%, and CO drops to ~7%. This pattern confirms that only basic pyrolysis (weak bond cleavage, decarboxylation, decarbonylation) occurs under inert conditions, with limited deep conversion. The gradual rise in N_2_ fraction is attributed to carrier gas dilution as active pyrolysis sites are consumed over time. In the CO_2_ atmosphere, CO becomes the dominant product with an extreme initial yield, reaching ~83% at 5 min, followed by a sharp decline, while CO_2_ content rises correspondingly. This trend is driven by the Boudouard reaction, where reactive char generated during pyrolysis reacts with CO_2_ to produce massive CO [44]. As the reaction time extends, active sites on the char surface are gradually consumed, and partial secondary oxidation of CO occurs, leading to a continuous drop in CO fraction and recovery of CO_2_, reflecting a highly dynamic and volatile reaction process. DFG and WFG atmospheres exhibit similar, moderate, and stable gas conversion behaviors, distinct from both inert N_2_ and highly reactive CO_2_ conditions. For DFG, CO_2_ remains the main component (~68% at 5 min, ~35% at 20 min), N_2_ content stabilizes at ~50% after 10 min, CO content stays low and decreases slightly over time, and trace O_2_ is rapidly consumed in the initial stage. WFG shows a highly consistent trend: CO_2_ dominates (~56–58%), N_2_ stabilizes at ~33–37%, and CO remains low (~5–7%). The mild reactivity of flue gas atmospheres stems from their composition (N_2_, CO_2_, trace O_2_), which promotes the cracking and conversion of pyrolysis intermediates without triggering excessive gas-phase reactions, resulting in a balanced and controllable reaction regime. The similarity between DFG and WFG confirms the universal regulatory effect of flue gas on biomass torrefaction [34,45].

## 4. Conclusions

This study systematically investigated the torrefaction behavior of demineralized poplar wood under N_2_, CO_2_, DFG, and WFG atmospheres, focusing on the effects of atmosphere composition and reaction time on pyrolysis kinetics, product distribution, and the physicochemical evolutions. The results demonstrate that the reaction atmosphere profoundly influences the thermal conversion of biomass.

Kinetic analysis reveals that the activation energy does not change monotonically with the atmosphere oxidizability. CO_2_ exhibits the highest *E_α_*, while the introduction of O_2_ and H_2_O in flue gas significantly lowers the energy barrier, with WFG giving the lowest *E_α_*. Correspondingly, the reaction mechanism shifts from diffusion control under N_2_ to nucleation and growth under CO_2_ and flue gas atmospheres, indicating that reactive components alter the rate-limiting step. This kinetic transition is consistent with the observed product distribution: WFG produces the lowest char yield and the highest gas yield. The effect of reaction time on gas yield is also more pronounced under flue gas, reflecting the cumulative nature of gasification reactions.

Regarding char properties, prolonged torrefaction consistently promotes deoxygenation and aromatization across all atmospheres, as shown by the increase in C_I_ (C–C, C=C, and C–H) and C_II_ (C–O/C–O–C). However, the extent of these changes varies with atmosphere: CO_2_ leads to more oxygenated intermediates, while WFG facilitates the deepest deoxygenation and the highest degree of carbon ordering. In contrast, pore structure development is modest under all conditions, suggesting that torrefaction under these mild conditions primarily modifies surface chemistry rather than porosity. Tar composition evolves from ketones toward aldehydes and saccharides over time, and this shift is accelerated under flue gas atmospheres, which correlates with the reduced activation energy and nucleation-dominated kinetics. Gas composition is atmosphere-dependent: CO_2_ dominates under N_2_, CO under CO_2_, while flue gas atmospheres yield moderate and stable gas profiles without excessive secondary reactions.

Overall, flue gas torrefaction, especially under WFG, effectively enhances biomass depolymerization by lowering the reaction energy barrier, promoting char deoxygenation and aromatization. These findings provide a scientific basis for process optimization and industrial integration of flue gas torrefaction. Further studies are recommended to directly validate the proposed reaction pathways, particularly the synergistic roles of O_2_ and H_2_O in radical-mediated and gasification reactions.

## Figures and Tables

**Table 1:** Proximate analysis of the raw and demineralized biomass (Wt. %, dry basis).

	Fixed Carbon	Volatile	Ash
Raw biomass	11.42	83.01	5.57
Demineralized biomass	17.27	82.70	0.03

**Table 2:** Key kinetic results calculated by the CR method.

Atmosphere	N_2_	CO_2_	DFG	WFG
Reaction mechanism	Three-dimensional diffusion (Z-L-T)	Avrami–Erofeeve	Avrami–Erofeeve	Avrami–Erofeeve
Model	D5	A1/4	A1/3	A1/3
g(α)	[(1 − α)^−1/3^ − 1]^2^	[−ln(1 − α)]^4^	[−ln(1 − α)]^3^	[−ln(1 − α)]^3^
Average E	176.95	313.93	133.34	128.23

**Table 3:** Relative content of carbon functional groups in biochar determined by XPS.

	C_I_	C_II_	C_III_	C_IV_
N_2_-5	49.92	40.72	6.09	3.28
N_2_-10	53.19	38.43	4.70	3.68
N_2_-20	60.43	33.55	3.73	2.29
CO_2_-5	48.77	33.57	12.81	7.85
CO_2_-10	55.72	30.70	8.70	4.88
CO_2_-20	63.14	29.76	4.04	3.06
DFG-5	47.6	41.60	7.62	3.18
DFG-10	52.28	39.65	5.01	3.07
DFG-20	64.25	24.99	5.30	5.46
WFG-5	52.07	40.72	4.36	2.85
WFG-10	61.45	30.02	3.62	4.91
WFG-20	61.92	29.31	3.94	4.82

**Table 4:** Pore structure characteristics of biochar measured by BET analysis.

Sample	Specific Surface Area (m^2^/g)	Average Pore Size (nm)
N_2_-5	1.463 ± 0.062	5.106 ± 0.125
N_2_-10	1.848 ± 0.071	8.920 ± 0.153
N_2_-20	2.531 ± 0.058	13.308 ± 0.182
CO_2_-5	1.562 ± 0.069	8.993 ± 0.142
CO_2_-10	2.457 ± 0.083	12.512 ± 0.165
CO_2_-20	5.139 ± 0.094	12.715 ± 0.172
DFG-5	2.624 ± 0.065	7.342 ± 0.131
DFG-10	3.312 ± 0.079	13.755 ± 0.188
DFG-20	4.638 ± 0.102	12.359 ± 0.153
WFG-5	3.916 ± 0.073	9.974 ± 0.146
WFG-10	5.360 ± 0.091	11.391 ± 0.163
WFG-20	5.487 ± 0.098	9.586 ± 0.136

## Data Availability

The original contributions presented in this study are included in the article/Supplementary Materials. Further inquiries can be directed to the corresponding authors.

## Supplementary Materials

The following supporting information can be downloaded at: https://www.mdpi.com/article/10.3390/polym18111370/s1, Figure S1: FWO plots of ln(*β_i_*) and 1000/*T_αi_* at different conversion rates; Figure S2: KAS plots of ln*β_i_*/*T*^2^*_αi_* and 1000/*T_αi_* at different conversion rates; Table S1: Kinetic parameters calculated by the FWO method; Table S2: Kinetic parameters calculated by the KAS method; Table S3: Common reaction mechanism models used in CR kinetic method and their differential f(α) and integral g(α) forms; Table S4: Kinetic parameters calculated by the CR method using 29 reaction models for the N_2_ atmosphere; Table S5: Kinetic parameters calculated by the CR method using 29 reaction models for the CO_2_ atmosphere; Table S6: Kinetic parameters calculated by the CR method using 29 reaction models for the DFG atmosphere; Table S7: Kinetic parameters calculated by the CR method using 29 reaction models for the WFG atmosphere.

## Abbreviations

The following abbreviations are used in this manuscript:

DFG	Dry flue gas
WFG	Wet flue gas
FWO	Flynn–Wall–Ozawa
KAS	Kissinger–Akahira–Sunose
CR	Coats–Redfern
BET	Brunauer–Emmett–Teller
DTG	Differential thermogravimetry
FTIR	Fourier-transform infrared spectroscopy
GC	Gas chromatography
GC/MS	Gas chromatography–mass spectrometry
TG	Thermogravimetry
TGA	Thermogravimetric analysis
XPS	X-ray photoelectron spectroscopy
