# Supplementary material for: Torrefaction of Demineralized Wood with Flue Gas: Kinetics, Product Distribution, and Thermal Conversion
Source: Polymers (Basel). 2026 May 31;18(11):1370. doi: 10.3390/polym18111370 (PMC13259145; doi:10.3390/polym18111370)
Supplement: Supplementary file 1 [file polymers-18-01370-s001.zip › polymers-4291170-supplementary.pdf]

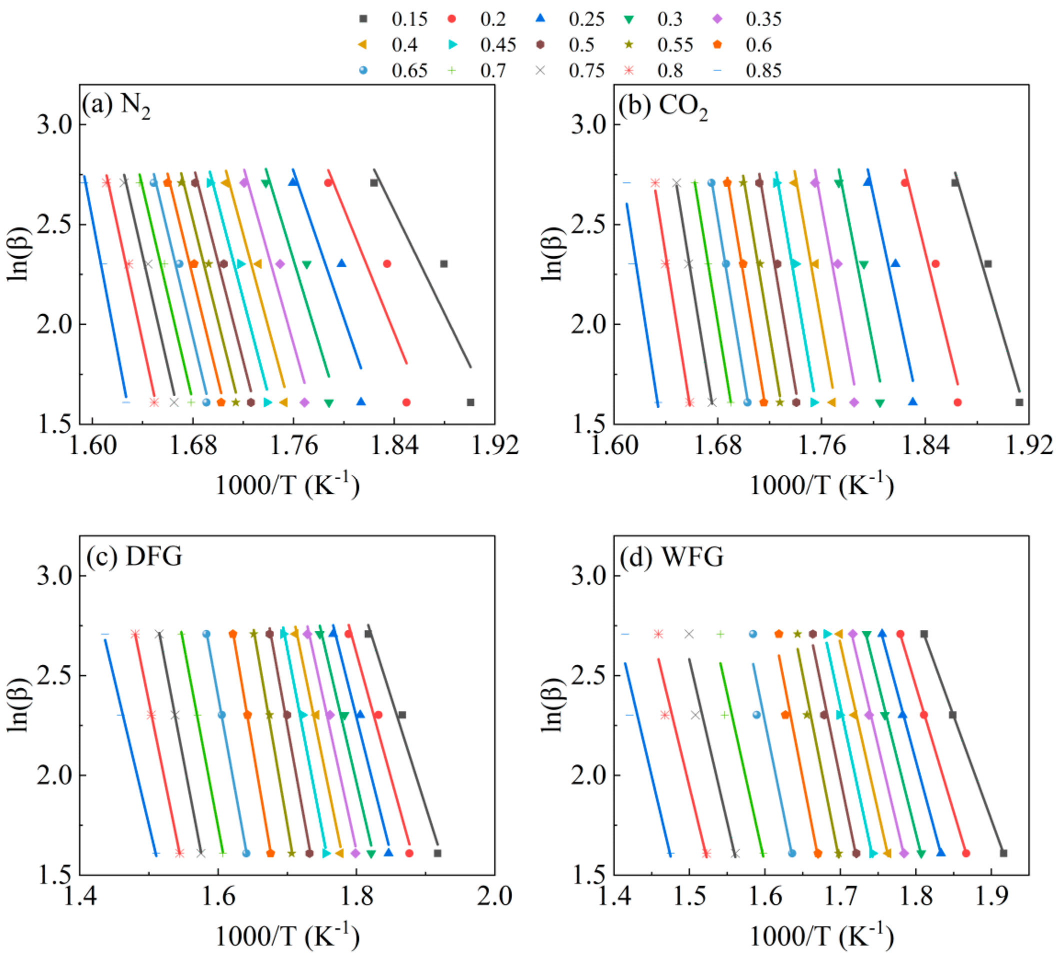

Figure S1. FWO plots of $ln(\beta_i)$ and $1000/T_{\alpha i}$ at different conversion rates

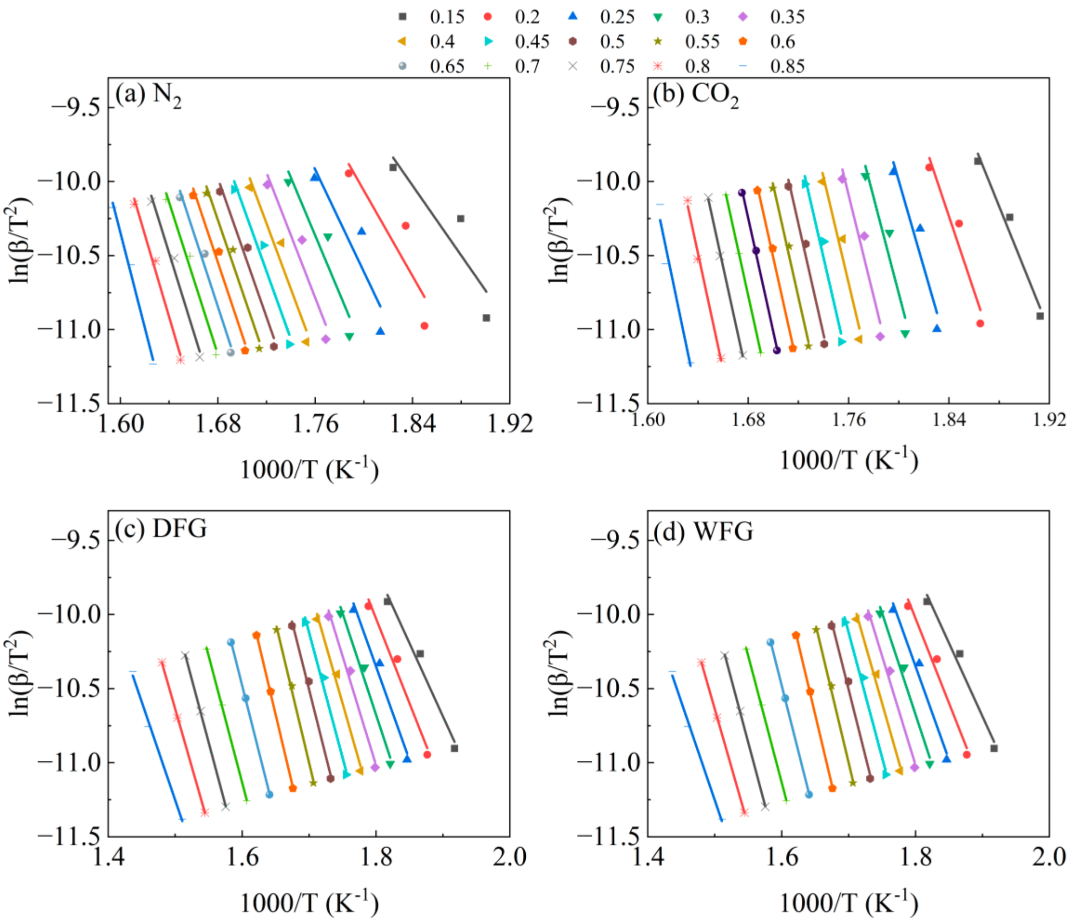

Figure S2. KAS plots of $ln\beta_i/T^2_{\alpha i}$ and $1000/T_{\alpha i}$ at different conversion rates.

Table S1. Kinetic parameters calculated by the FWO method

α	N_2			CO_2			DFG			WFG		
	E(kJ/mol)	A(min^{-1})	R^2	E(kJ/mol)	A(min^{-1})	R^2	E(kJ/mol)	A(min^{-1})	R^2	E(kJ/mol)	A(min^{-1})	R^2
0.15	101.48	7.19e8	0.85	173.77	1.83e16	0.97	86.27	2.26e7	0.98	81.98	7.89e6	1.00
0.2	122.33	5.80e10	0.82	207.56	2.29e19	0.94	98.59	3.27e8	0.98	98.97	3.01e8	1.00
0.25	144.74	6.13e12	0.85	239.61	1.80e22	0.92	108.18	2.49e9	0.98	110.36	3.23e9	1.00
0.3	163.17	2.66e14	0.90	263.48	2.22e24	0.93	116.64	1.43e10	0.98	118.75	1.76e10	1.00
0.35	175.36	3.00e15	0.93	279.96	5.36e25	0.94	124.91	7.60e10	0.99	125.74	6.94e10	1.00
0.4	183.29	1.37e16	0.95	291.02	4.00e26	0.96	133.32	3.99e11	0.99	132.28	2.41e11	0.99
0.45	189.27	4.13e16	0.96	298.54	1.40e27	0.97	142.15	2.16e12	0.99	138.84	8.08e11	0.99
0.5	194.24	1.02e17	0.97	303.45	2.80e27	0.98	150.72	1.02e13	0.99	145.32	2.52e12	0.99
0.55	198.80	2.28e17	0.98	306.38	3.70e27	0.99	158.13	3.39e13	1.00	151.03	6.21e12	0.98
0.6	202.96	4.67e17	0.98	308.93	4.47e27	0.99	159.34	2.68e13	1.00	153.59	7.14e12	0.95
0.65	207.26	9.73e17	0.98	311.60	5.49e27	1.00	150.38	2.47e12	1.00	146.52	1.02e12	0.92
0.7	211.58	1.99e18	0.98	312.07	4.12e27	1.00	144.53	4.67e11	1.00	134.48	5.40e10	0.92
0.75	217.98	5.99e18	0.99	314.20	4.16e27	1.00	143.24	2.35e11	1.00	127.91	9.41e9	0.94
0.8	229.05	4.31e19	0.99	318.66	6.08e27	0.99	134.45	2.93e10	1.00	122.45	2.14e9	0.94
0.85	261.80	2.00e22	0.99	330.00	2.70e28	0.96	115.16	5.53e8	1.00	126.45	2.47e9	0.92
Average	186.89	-	0.94	283.95	-	0.97	131.07	-	0.99	127.64	-	0.97

Table S2. Kinetic parameters calculated by the KAS method

α	N_2			CO_2			DFG			WFG		
	E(kJ/mol)	A(min^{-1})	R^2	E(kJ/mol)	A(min^{-1})	R^2	E(kJ/mol)	A(min^{-1})	R^2	E(kJ/mol)	A(min^{-1})	R^2
0.15	98.23	2.34e8	0.82	174.69	1.87e16	0.97	82.19	5.27e6	0.98	77.66	1.65e6	1.00
0.2	120.03	2.66e10	0.80	210.17	3.21e19	0.94	95.04	9.74e7	0.98	95.40	8.94e7	1.00
0.25	143.53	3.84e12	0.84	243.85	3.26e22	0.92	105.03	8.73e8	0.98	107.28	1.17e9	1.00
0.3	162.87	2.08e14	0.89	268.94	4.76e24	0.92	113.85	5.72e9	0.98	116.02	7.22e9	1.00
0.35	175.65	2.66e15	0.93	286.24	1.27e26	0.94	122.48	3.42e10	0.98	123.29	3.12e10	1.00
0.4	183.94	1.30e16	0.95	297.83	1.01e27	0.95	131.26	2.01e11	0.99	130.09	1.18e11	0.99
0.45	190.17	4.14e16	0.96	305.70	3.65e27	0.97	140.47	1.21e12	0.99	136.91	4.26e11	0.99
0.5	195.36	1.06e17	0.97	310.81	7.45e27	0.98	149.41	6.32e12	0.99	143.64	1.43e12	0.98
0.55	200.10	2.45e17	0.97	313.84	9.87e27	0.99	157.09	2.24e13	1.00	149.54	3.71e12	0.97
0.6	204.43	5.18e17	0.98	316.46	1.20e28	0.99	158.18	1.73e13	1.00	152.09	4.27e12	0.95
0.65	208.91	1.11e18	0.98	319.21	1.47e28	1.00	148.49	1.36e12	1.00	144.41	5.29e11	0.91
0.7	213.40	2.33e19	0.98	319.63	1.09e28	1.00	142.09	2.26e11	1.00	131.42	2.22e10	0.91
0.75	220.09	7.35e18	0.98	321.79	1.10e28	1.00	140.50	1.07e11	1.00	124.21	3.32e9	0.93
0.8	231.67	5.74e19	0.99	326.40	1.62e28	0.99	130.99	1.12e10	1.00	118.17	6.52e8	0.93
0.85	266.13	3.42e22	0.99	338.23	7.49e28	0.95	110.34	1.44e8	0.99	122.03	7.54e8	0.91
Average	187.63	-	0.94	290.25	-	0.97	128.49	-	0.99	124.81	-	0.96

Table S3. Common reaction mechanism models used in CR kinetic method and their differential f(α) and integral g(α) forms

Chemical reaction	Model	f(α)	g(α)
Fn-Chemical reaction model	n=0	1	α
Fn-Chemical reaction model	n=1/2	$(1-\alpha)^{1/2}$	$[(1-\alpha)^{1/2}-1]/(-1/2)$
Fn-Chemical reaction model	n=1	$(1-\alpha)$	$-\ln(1-\alpha)$
Fn-Chemical reaction model	n=3/2	$(1-\alpha)^{3/2}$	$[(1-\alpha)^{-1/2}-1]/(1/2)$
Fn-Chemical reaction model	n=2	$(1-\alpha)^{2}$	$(1-\alpha)^{-1}-1$
Fn-Chemical reaction model	n=5/2	$(1-\alpha)^{5/2}$	$[(1-\alpha)^{-3/2}-1]/(3/2)$
Fn-Chemical reaction model	n=3	$(1-\alpha)^{3}$	$[(1-\alpha)^{-2}-1]/2$
An - Avrami - Erofeeve	n=1/4	$1/4(1-\alpha)[-\ln(1-\alpha)]^{-3}$	$[-\ln(1-\alpha)]^{4}$
An - Avrami - Erofeeve	n=1/3	$1/3(1-\alpha)[-\ln(1-\alpha)]^{-2}$	$[-\ln(1-\alpha)]^{3}$
An - Avrami - Erofeeve	n=1/2	$1/2(1-\alpha)[-\ln(1-\alpha)]^{-1}$	$[-\ln(1-\alpha)]^{2}$
An - Avrami - Erofeeve	n=2/3	$2/3(1-\alpha)[-\ln(1-\alpha)]^{-1/2}$	$[-\ln(1-\alpha)]^{3/2}$
An - Avrami - Erofeeve	n=4/3	$4/3(1-\alpha)[-\ln(1-\alpha)]^{1/4}$	$[-\ln(1-\alpha)]^{3/4}$
An - Avrami - Erofeeve	n=3/2	$3/2(1-\alpha)[-\ln(1-\alpha)]^{1/3}$	$[-\ln(1-\alpha)]^{2/3}$
An - Avrami - Erofeeve	n=2	$2(1-\alpha)[-\ln(1-\alpha)]^{1/2}$	$[-\ln(1-\alpha)]^{1/2}$
An - Avrami - Erofeeve	n=5/2	$5/2(1-\alpha)[-\ln(1-\alpha)]^{3/5}$	$[-\ln(1-\alpha)]^{2/5}$
An - Avrami - Erofeeve	n=3	$3(1-\alpha)[-\ln(1-\alpha)]^{2/3}$	$[-\ln(1-\alpha)]^{1/3}$
An - Avrami - Erofeeve	n=4	$4(1-\alpha)[-\ln(1-\alpha)]^{3/4}$	$[-\ln(1-\alpha)]^{1/4}$
Pn - power-law	n=1/2	$1/2\alpha^{-1}$	α^{2}
Pn - power-law	n=2/3	$2/3\alpha^{-1/2}$	$\alpha^{3/2}$
Pn - power-law	n=2	$2\alpha^{1/2}$	$\alpha^{1/2}$
Pn - power-law	n=3	$3\alpha^{2/3}$	$\alpha^{1/3}$
Pn - power-law	n=4	$4\alpha^{3/4}$	$\alpha^{1/4}$
Rn - Phase interfacial reaction	n=2 Shrinkage geometrical (cylindrical)	$2(1-\alpha)^{1/2}$	$1-(1-\alpha)^{1/2}$
Rn - Phase interfacial reaction	n=3 Shrinkage geometrical (spherical)	$3(1-\alpha)^{2/3}$	$1-(1-\alpha)^{1/3}$
One-dimensional diffusion	D1	$1/(2\alpha)$	α^{2}
Two-dimensional diffusion	D2	$[-\ln(1-\alpha)]^{-1}$	$[(1-\alpha)\ln(1-\alpha)]+\alpha$
Three-dimensional diffusion (Jander)	D3	$3/2(1-\alpha)^{2/3}[1-(1-\alpha)^{1/3}]^{-1}$	$[1-(1-\alpha)^{1/3}]^{2}$
Three-dimensional diffusion (Ginstling)	D4	$3/2[1-(1-\alpha)^{1/3}]^{-1}$	$1-2/3\alpha-(1-\alpha)^{2/3}$
Three-dimensional diffusion (Z-L-T)	D5	$3/2(1-\alpha)^{4/3}[(1-\alpha)^{-1/3}-1]^{-1}$	$[(1-\alpha)^{-1/3}-1]^{2}$

Table S4. Kinetic parameters calculated by the CR method using 29 reaction models for the N_2 atmosphere.

Model	5_E	10_E	20_E	Average E	5_R^2	10_R^2	20_R^2	Average R^2	5_lnA	10_lnA	20_lnA	Average lnA
F0	48.493	47.540	57.169	51.067	0.992	0.995	0.992	0.993	2.796	3.137	5.784	3.906
F1/2	58.644	57.540	69.055	61.746	0.993	0.995	0.997	0.995	5.285	5.569	8.565	6.473
F1	70.556	69.257	83.002	74.272	0.988	0.988	0.996	0.990	8.156	8.371	11.780	9.435
F2/3	84.284	82.746	99.071	88.700	0.978	0.977	0.989	0.981	11.420	11.550	15.438	12.803
F2	99.773	97.952	117.201	104.975	0.965	0.963	0.978	0.969	15.063	15.094	19.526	16.561
F5/2	116.874	114.734	137.221	122.943	0.952	0.949	0.967	0.956	19.051	18.971	24.005	20.676
F3	135.384	132.895	158.893	142.391	0.940	0.936	0.955	0.943	23.338	23.138	28.825	25.100
A1/4	310.586	305.710	361.257	325.851	0.989	0.990	0.996	0.992	58.487	57.391	68.507	61.462
A1/3	230.576	226.892	268.505	241.991	0.989	0.990	0.996	0.992	41.906	41.248	49.791	44.315
A1/2	150.566	148.075	175.753	158.131	0.989	0.989	0.996	0.991	25.197	24.976	30.949	27.040
A2/3	110.561	108.666	129.377	116.202	0.989	0.989	0.996	0.991	16.747	16.744	21.433	18.308
A4/3	50.554	49.553	59.814	53.307	0.987	0.987	0.995	0.990	3.752	4.074	6.847	4.891
A3/2	43.886	42.985	52.084	46.318	0.986	0.987	0.995	0.989	2.254	2.612	5.174	3.346
A2	30.551	29.849	36.626	32.342	0.984	0.985	0.995	0.988	-0.822	-0.394	1.752	0.179
A5/2	22.550	21.967	27.351	23.956	0.982	0.982	0.994	0.986	-2.754	-2.285	-0.382	-1.807
A3	17.216	16.712	21.167	18.365	0.979	0.979	0.993	0.984	-4.110	-3.615	-1.866	-3.197
A4	10.549	10.144	13.438	11.377	0.970	0.970	0.991	0.977	-5.957	-5.434	-3.855	-5.082
P1/2	106.439	104.640	124.089	111.723	0.993	0.996	0.994	0.994	14.880	14.913	19.355	16.383
P2/3	77.466	76.090	90.629	81.395	0.993	0.996	0.993	0.994	8.913	9.101	12.643	10.219
P2	19.520	18.990	23.710	20.740	0.987	0.992	0.988	0.989	-3.763	-3.275	-1.494	-2.844
P3	9.862	9.473	12.556	10.630	0.978	0.985	0.981	0.981	-6.329	-5.801	-4.263	-5.464
P4	5.033	4.715	6.980	5.576	0.952	0.966	0.964	0.961	-7.943	-7.415	-5.916	-7.091
R2	58.644	57.540	69.055	61.746	0.993	0.995	0.997	0.995	4.591	4.876	7.872	5.780
R3	62.414	61.250	73.469	65.711	0.992	0.993	0.997	0.994	5.100	5.363	8.489	6.317
D1	106.439	104.640	124.089	111.723	0.993	0.996	0.994	0.994	14.880	14.913	19.355	16.383
D2	118.961	116.983	138.750	124.898	0.994	0.996	0.997	0.996	17.139	17.105	21.978	18.741
D3	134.282	132.060	156.689	141.010	0.993	0.994	0.998	0.995	19.215	19.091	24.497	20.934
D4	124.021	121.963	144.675	130.220	0.994	0.996	0.997	0.996	16.820	16.756	21.805	18.460
**D5**	**168.469**	**165.668**	**196.710**	**176.949**	**0.983**	**0.983**	**0.992**	**0.986**	**27.139**	**26.807**	**33.407**	**29.118**

Table S5. Kinetic parameters calculated by the CR method using 29 reaction models for the CO_2 atmosphere.

Model	5_E	10_E	20_E	Average E	5_R^2	10_R^2	20_R^2	Average R^2	5_lnA	10_lnA	20_lnA	Average lnA
F0	47.154	46.605	51.395	48.385	0.990	0.985	0.987	0.988	2.578	3.035	4.719	3.444
F1/2	57.299	56.976	62.509	58.928	0.995	0.995	0.996	0.996	5.087	5.577	7.390	6.018
F1	69.238	69.217	75.597	71.350	0.993	0.998	0.998	0.996	7.988	8.522	10.483	8.998
F2/3	83.025	83.387	90.721	85.711	0.986	0.993	0.993	0.991	11.290	11.881	14.010	12.394
F2	98.606	99.427	107.819	101.950	0.975	0.985	0.985	0.982	14.981	15.639	17.955	16.192
F5/2	115.826	117.174	126.724	119.908	0.963	0.976	0.975	0.971	19.024	19.761	22.280	20.355
F3	134.474	136.406	147.203	139.361	0.952	0.966	0.965	0.961	23.373	24.196	26.936	24.835
**A1/4**	**305.163**	**305.434**	**331.184**	**313.927**	**0.994**	**0.998**	**0.998**	**0.997**	**57.840**	**57.973**	**63.516**	**59.777**
A1/3	226.521	226.695	245.988	233.068	0.994	0.998	0.998	0.997	41.419	41.686	46.034	43.046
A1/2	147.879	147.956	160.792	152.209	0.994	0.998	0.998	0.996	24.870	25.270	28.423	26.188
A2/3	108.559	108.586	118.195	111.780	0.993	0.998	0.998	0.996	16.499	16.967	19.523	17.663
A4/3	49.577	49.532	54.298	51.136	0.992	0.997	0.997	0.996	3.623	4.190	5.856	4.556
A3/2	43.024	42.971	47.198	44.397	0.992	0.997	0.997	0.996	2.138	2.715	4.284	3.046
A2	29.917	29.847	32.999	30.921	0.991	0.997	0.997	0.995	-0.913	-0.314	1.062	-0.055
A5/2	22.053	21.974	24.479	22.835	0.990	0.996	0.997	0.994	-2.830	-2.219	-0.955	-2.002
A3	16.810	16.724	18.799	17.445	0.988	0.996	0.996	0.993	-4.176	-3.558	-2.365	-3.367
A4	10.256	10.163	11.700	10.706	0.983	0.994	0.995	0.990	-6.014	-5.389	-4.271	-5.225
P1/2	103.711	102.732	112.390	106.278	0.992	0.988	0.989	0.990	14.464	14.724	17.308	15.499
P2/3	75.433	74.668	81.893	77.331	0.992	0.987	0.989	0.989	8.597	8.956	11.088	9.547
P2	18.875	18.541	20.898	19.438	0.984	0.976	0.979	0.980	-3.886	-3.336	-2.084	-3.102
P3	9.449	9.187	10.732	9.789	0.971	0.955	0.964	0.963	-6.428	-5.854	-4.718	-5.667
P4	4.735	4.510	5.649	4.965	0.935	0.895	0.926	0.919	-8.043	-7.474	-6.344	-7.287
R2	57.299	56.976	62.509	58.928	0.995	0.995	0.996	0.996	4.394	4.884	6.697	5.325
R3	61.074	60.843	66.646	62.855	0.995	0.997	0.997	0.997	4.911	5.414	7.275	5.866
D1	103.711	102.732	112.390	106.278	0.992	0.988	0.989	0.990	14.464	14.724	17.308	15.499
D2	116.209	115.488	126.074	119.257	0.995	0.994	0.995	0.995	16.742	17.033	19.784	17.853
D3	131.553	131.209	142.892	135.218	0.996	0.997	0.998	0.997	18.850	19.193	22.140	20.061
D4	121.276	120.677	131.627	124.527	0.996	0.996	0.996	0.996	16.432	16.741	19.557	17.577
D5	165.854	166.423	180.508	170.929	0.990	0.996	0.996	0.994	26.858	27.328	30.703	28.297

Table S6. Kinetic parameters calculated by the CR method using 29 reaction models for the DFG atmosphere.

Model	5_E	10_E	20_E	Average E	5_R^2	10_R^2	20_R^2	Average R^2	5_lnA	10_lnA	20_lnA	Average lnA
F0	23.460	21.807	22.326	22.531	0.864	0.812	0.771	0.816	-3.231	-3.094	-2.379	-2.901
F1/2	30.207	28.629	29.528	29.454	0.923	0.886	0.855	0.888	-1.410	-1.270	-0.495	-1.058
F1	38.239	36.810	38.201	37.750	0.960	0.936	0.913	0.937	0.677	0.826	1.676	1.060
F2/3	47.598	46.398	48.398	47.464	0.982	0.967	0.951	0.967	3.040	3.205	4.146	3.463
F2	58.241	57.346	60.065	58.551	0.992	0.984	0.973	0.983	5.669	5.856	6.905	6.143
F5/2	70.055	69.528	73.065	70.883	0.996	0.992	0.985	0.991	8.540	8.754	9.924	9.073
F3	82.885	82.772	87.207	84.288	0.996	0.996	0.991	0.994	11.619	11.864	13.167	12.216
A1/4	182.264	177.448	183.669	181.127	0.973	0.957	0.940	0.957	30.693	29.519	30.850	30.354
**A1/3**	**134.256**	**130.568**	**135.180**	**133.335**	**0.972**	**0.956**	**0.938**	**0.955**	**20.903**	**20.172**	**21.342**	**20.806**
A1/2	86.247	83.689	86.690	85.542	0.969	0.952	0.933	0.951	10.975	10.688	11.696	11.120
A2/3	62.243	60.250	62.446	61.646	0.967	0.947	0.927	0.947	5.907	5.839	6.768	6.171
A4/3	26.237	25.090	26.079	25.802	0.952	0.923	0.896	0.923	-2.071	-1.817	-1.006	-1.631
A3/2	22.236	21.184	22.038	21.819	0.946	0.914	0.885	0.915	-3.027	-2.739	-1.942	-2.569
A2	14.235	13.371	13.957	13.854	0.925	0.880	0.843	0.883	-5.054	-4.706	-3.932	-4.564
A5/2	9.434	8.683	9.108	9.075	0.892	0.824	0.777	0.831	-6.413	-6.042	-5.279	-5.911
A3	6.233	5.557	5.875	5.889	0.833	0.728	0.671	0.744	-7.460	-7.091	-6.331	-6.961
A4	2.233	1.651	1.835	1.906	0.516	0.284	0.252	0.351	-9.277	-9.058	-8.261	-8.866
P1/2	56.688	53.683	54.939	55.103	0.906	0.870	0.839	0.872	3.716	3.450	4.206	3.791
P2/3	40.074	37.745	38.632	38.817	0.894	0.854	0.820	0.856	0.337	0.276	1.012	0.541
P2	6.845	5.869	6.019	6.244	0.671	0.541	0.483	0.565	-7.496	-7.228	-6.532	-7.085
P3	1.307	0.556	0.583	0.816	0.136	0.022	0.019	0.059	-10.162	-10.525	-9.813	-10.167
P4	-1.462	-2.100	-2.134	-1.899	0.248	0.351	0.300	0.300	-10.556	-9.667	-8.989	-9.737
R2	30.207	28.629	29.528	29.454	0.923	0.886	0.855	0.888	-2.103	-1.963	-1.188	-1.752
R3	32.738	31.201	32.251	32.063	0.937	0.906	0.877	0.907	-1.844	-1.701	-0.903	-1.483
D1	56.688	53.683	54.939	55.103	0.906	0.870	0.839	0.872	3.716	3.450	4.206	3.791
D2	64.950	62.004	63.706	63.553	0.930	0.901	0.874	0.902	5.087	4.803	5.623	5.171
D3	75.245	72.470	74.790	74.168	0.953	0.930	0.908	0.930	6.108	5.820	6.730	6.219
D4	68.346	65.454	67.358	67.053	0.939	0.912	0.886	0.912	4.419	4.134	4.983	4.512
D5	98.432	96.163	99.949	98.181	0.981	0.968	0.952	0.967	11.704	11.425	12.549	11.893

Table S7. Kinetic parameters calculated by the CR method using 29 reaction models for the WFG atmosphere.

Model	5_E	10_E	20_E	Average E	5_R^2	10_R^2	20_R^2	Average R^2	5_lnA	10_lnA	20_lnA	Average lnA
F0	21.903	20.272	21.457	21.211	0.843	0.778	0.748	0.790	-3.683	-3.570	-2.639	-3.297
F1/2	28.457	26.830	28.453	27.913	0.907	0.861	0.835	0.868	-1.902	-1.809	-0.802	-1.504
F1	36.297	34.706	36.876	35.960	0.950	0.918	0.897	0.922	0.143	0.209	1.310	0.554
F2/3	45.465	43.945	46.775	45.395	0.976	0.955	0.938	0.956	2.462	2.496	3.710	2.890
F2	55.918	54.502	58.101	56.174	0.990	0.976	0.964	0.977	5.045	5.043	6.389	5.492
F5/2	67.538	66.254	70.720	68.171	0.996	0.988	0.979	0.988	7.866	7.824	9.319	8.336
F3	80.163	79.032	84.447	81.214	0.998	0.994	0.988	0.993	10.892	10.806	12.463	11.387
A1/4	174.844	169.586	178.654	174.361	0.967	0.946	0.930	0.948	28.800	27.353	29.558	28.570
**A1/3**	**128.662**	**124.626**	**131.395**	**128.227**	**0.965**	**0.944**	**0.927**	**0.945**	**19.465**	**18.526**	**20.361**	**19.451**
A1/2	82.479	79.666	84.135	82.093	0.962	0.939	0.921	0.941	9.992	9.559	11.025	10.192
A2/3	59.388	57.186	60.506	59.027	0.958	0.933	0.914	0.935	5.150	4.968	6.250	5.456
A4/3	24.751	23.466	25.061	24.426	0.938	0.900	0.875	0.904	-2.496	-2.312	-1.299	-2.036
A3/2	20.903	19.719	21.123	20.582	0.931	0.888	0.862	0.894	-3.418	-3.195	-2.210	-2.941
A2	13.206	12.226	13.246	12.893	0.903	0.840	0.810	0.851	-5.382	-5.093	-4.159	-4.878
A5/2	8.587	7.730	8.520	8.279	0.855	0.760	0.729	0.782	-6.715	-6.404	-5.489	-6.203
A3	5.509	4.733	5.370	5.204	0.771	0.624	0.601	0.666	-7.761	-7.462	-6.543	-7.255
A4	1.660	0.986	1.432	1.359	0.337	0.108	0.154	0.199	-9.712	-9.741	-8.606	-9.353
P1/2	53.691	50.797	53.298	52.595	0.893	0.851	0.825	0.856	2.920	2.626	3.754	3.100
P2/3	37.797	35.535	37.378	36.903	0.879	0.831	0.803	0.837	-0.285	-0.370	0.657	0.001
P2	6.009	5.009	5.537	5.518	0.601	0.447	0.430	0.492	-7.830	-7.607	-6.735	-7.390
P3	0.711	-0.079	0.230	0.287	0.042	0.000	0.003	0.015	-10.916	-12.638	-10.828	-11.461
P4	-1.938	-2.623	-2.423	-2.328	0.355	0.440	0.344	0.380	-10.388	-9.573	-8.932	-9.631
R2	28.457	26.830	28.453	27.913	0.907	0.861	0.835	0.868	-2.595	-2.502	-1.496	-2.198
R3	30.924	29.305	31.098	30.442	0.923	0.883	0.858	0.888	-2.349	-2.264	-1.229	-1.947
D1	53.691	50.797	53.298	52.595	0.893	0.851	0.825	0.856	2.920	2.626	3.754	3.100
D2	61.698	58.792	61.815	60.768	0.919	0.883	0.859	0.887	4.231	3.893	5.111	4.412
D3	71.733	68.864	72.579	71.059	0.944	0.915	0.894	0.918	5.188	4.804	6.141	5.378
D4	65.007	62.112	65.362	64.160	0.928	0.895	0.872	0.898	3.541	3.189	4.445	3.725
D5	94.410	91.684	97.008	94.367	0.976	0.957	0.942	0.958	10.657	10.173	11.785	10.872
